# Evaluation of Dried Blood and Cerebrospinal Fluid Filter Paper Spots for Storing and Transporting Clinical Material for the Molecular Diagnosis of Invasive Meningococcal Disease

**DOI:** 10.3390/ijms231911879

**Published:** 2022-10-06

**Authors:** Brenda A. Kwambana-Adams, Stephen A. Clark, Nicole Tay, Schadrac Agbla, Chrispin Chaguza, Eunice W. Kagucia, Ray Borrow, Robert S. Heyderman

**Affiliations:** 1 NIHR Global Health Research Unit on Mucosal Pathogens, Division of Infection and Immunity, University College London, London WC1E 6BT, UK; 2 Clinical Sciences Department, Liverpool School of Tropical Medicine, Liverpool L3 5QA, UK; 3 Malawi-Liverpool-Wellcome Clinical Research Programme (MLW), Blantyre P.O. Box 30096, Malawi; 4 Meningococcal Reference Unit, United Kingdom Health Security Agency (UKHSA), Manchester M13 9WL, UK; 5 Department of Health Data Science, University of Liverpool, Liverpool L69 3GF, UK; 6 Department of Infectious Disease Epidemiology, London School of Hygiene and Tropical Medicine, London WC1E 7HT, UK; 7 Wellcome Sanger Institute, Wellcome Genome Campus, Hinxton, Cambridge CB10 1SA, UK; 8 Department of Epidemiology of Microbial Diseases, Yale School of Public Health, Yale University, New Haven, CT 06510, USA; 9 Department of Epidemiology and Demography, KEMRI-Wellcome Trust Research Programme, Kilifi P.O. Box 230-8010, Kenya

**Keywords:** *Neisseria meningitidis*, invasive meningococcal disease, meningitis, bacteraemia, dried filter paper spot, cerebrospinal fluid, blood, real-time PCR, diagnosis, whole genome sequencing, targeted enrichment

## Abstract

To improve the storage and transport of clinical specimens for the diagnosis of *Neisseria meningitidis* (Nm) infections in resource-limited settings, we have evaluated the performance of dried blood spot (DBS) and dried cerebrospinal fluid spot (DCS) assays. DBS and DCS were prepared on filter paper from liquid specimens previously tested for Nm in the United Kingdom. Nm was detected and genogrouped by real-time PCR performed on crude genomic DNA extracted from the DBS (*n* = 226) and DCS (*n* = 226) specimens. Targeted whole-genome sequencing was performed on a subset of specimens, DBS (*n* = 4) and DCS (*n* = 6). The overall agreement between the analysis of liquid and dried specimens was (94.2%; 95% CI 90.8–96.7) for blood and (96.4%; 95% CI 93.5–98.0) for cerebrospinal fluid. Relative to liquid specimens as the reference, the DBS and DCS assays had sensitivities of (89.1%; 95% CI 82.7–93.8) and (94.2%; 95% CI 88.9–97.5), respectively, and both assays had specificities above 98%. A genogroup was identified by dried specimen analysis for 81.9% of the confirmed meningococcal infections. Near full-length Nm genome sequences (>86%) were obtained for all ten specimens tested which allowed determination of the sequence type, clonal complex, presence of antimicrobial resistance and other meningococcal genotyping. Dried blood and CSF filter spot assays offer a practical alternative to liquid specimens for the molecular and genomic characterisation of invasive meningococcal diseases in low-resource settings.

## 1. Introduction

*Neisseria meningitidis* (Nm) is a commensal of the human pharynx which can invade the bloodstream and meninges to cause sepsis and/or meningitis, respectively [1]. Although there are twelve Nm serogroups based on capsular polysaccharide structure, serogroups A (NmA), B (NmB), C (NmC), W (NmW), X (NmX) and Y (NmY) account for almost all invasive meningococcal disease (IMD) globally [2].

For over 100 years, large populations within the so-called “African meningitis belt” have experienced periodic devastating and often protracted IMD outbreaks. NmA strains were the dominant cause of meningitis outbreaks prior to the roll-out of the meningococcal A protein-polysaccharide conjugate vaccine (MenAfriVac©) mass vaccination campaigns across 24 countries in the African meningitis belt between 2010 and 2018 [3]. To maintain high levels of herd immunity and sustain control of NmA, more than half of the 26 African meningitis belt countries have introduced MenAfriVac© into their infant immunisation programmes. However, hypervirulent NmC, NmW and NmX strains with epidemic potential have replaced NmA as the predominant cause of IMD in the African meningitis belt [4,5,6,7]. Currently, available polyvalent meningococcal vaccines are not affordable and have limited stockpiles, hence, their use in the African meningitis belt is restricted to reactive vaccination campaigns during suspected outbreaks [8]. A pentavalent meningococcal conjugate vaccine targeting NmA, NmC, NmY, NmW and NmX is expected to become available in 2022 and post-implementation monitoring of IMD will be crucial in the African meningitis belt [9].

During suspected IMD outbreaks, prompt laboratory confirmation and characterisation of meningococcal disease cases are essential to inform public health decisions, especially reactive vaccination. The WHO guidelines for the management of meningitis outbreaks recommend confirmation of meningococcal meningitis by the detection of bacteria in the cerebrospinal fluid (CSF) or blood of a suspected case [10]. However, across the meningitis belt, laboratory confirmation of IMD is often hindered by low CSF specimen collection rates, low sensitivity of culture-based identification of Nm coupled with poor bacteriologic diagnostic capacity at health facilities. Furthermore, there is often a lack of resources and infrastructure for cold chain storage and transport of clinical specimens or isolates to national or regional public health laboratories for further molecular analysis. The 6th Strategic Goal of The World Health Organization (WHO) Roadmap to Defeating Meningitis by 2030 focuses on the need to improve the diagnosis of meningitis at all levels of care [11]. An objective outlined in Strategic Goal 6 of the roadmap is to evaluate the role of blood sampling and dried bloodspots on filter paper in diagnosing meningitis or sepsis by 2022 in low- and middle-income countries (LMICs), especially in the African meningitis belt [11].

Procedures utilising dried blood spots (DBS), whereby small volumes of blood are inoculated and dried onto filter paper, may offer an inexpensive and practical approach for the stable storage of nucleic acids at ambient temperature. Such procedures have been shown to successfully facilitate the detection of a range of diseases including HIV, hepatitis, dengue, syphilis, and malaria [12,13]. Although dried CSF spots (DCS) have been much less frequently utilised, the use of a DBS assay for *Streptococcus pneumoniae* infections [14,15] and bacterial meningitis [16] have shown promising results, including in low-resource settings.

In this study, we evaluated new dried filter spot methods for both blood and CSF, leveraging well-characterised specimens archived at the United Kingdom Health Security Agency (UKHSA) Meningococcal Reference Unit (MRU). These methods enable storage and transportation of specimens at ambient temperature, thereby eliminating the need for a cold chain. These methods utilise a crude nucleic acid extraction procedure for blood and CSF specimens which is quick, affordable and uses fewer reagents and consumables compared to traditional column-based extraction methods [17]. These highly sensitive, specific, and affordable meningitis diagnostic methods that allow small volumes of clinical specimens to be stored for extended periods at room temperature while awaiting transport to central laboratories could lead to significant improvement in IMD case ascertainment in LMICs.

## 2. Results

Blood (*n* = 226) and CSF (*n* = 226) specimens collected from 537 patients in England between 2007 and 2020 were studied to evaluate DBS and DCS assays for the diagnosis of IMD. Twelve patients had paired blood and CSF specimens collected, and three patients had the same sample type collected on different days. For comparison, a total of 138 DBS and 138 DCS specimens were prepared from liquid specimens that had previously tested positive for Nm by real-time PCR. Likewise, 138 DBS and 138 DCS specimens were prepared from specimens that had previously tested negative for Nm by real-time PCR.

### 2.1. Sensitivity and Specificity of the Dried Spot Assay

There was good concordance between the real-time PCR cycle thresholds (CTs) for the detection and genogrouping of Nm using the standard testing performed on liquid specimens and dried spot testing (Supplemental Figure S1). Although the CTs for Nm detection were generally higher in liquid specimens, the correlations with the dried specimen assay were strong for DBS and DCS, *p* = 0.90 and *p* = 0.76, respectively. For the genogrouping assays, there appeared to be more variability in the CTs between the liquid and dried assays with correlation coefficients of *p* = 0.61 for both blood and CSF specimens. The total agreement between the liquid and dried extracts for detection of Nm was high: (94.2%; 95% CI 90.8–96.7) and (96.4%; 95% CI 93.4–98.2) for blood and CSF specimens, respectively (Table 1). The dried filter spot assay was highly specific for both blood at (99.3%; 95% CI 96.00–99.9) and CSF at (98.6%; 95% CI 94.9–99.8). Nm was detected and genogrouped using the dried filter spot assay in 3/276 specimens that had previously tested negative in the original analysis of the liquid specimens. The dried spot assay had high sensitivity, (94.2%; 95% CI 90.8–96.7) for the detection of Nm in CSF specimens while the sensitivity of the DBS assay was lower at (89.1%; 95% CI 82.7–93.8) (Table 1).

We also applied a more stringent analytic cut-off of CT < 36 to determine confirmed cases in the liquid specimen analysis. An analytic cut-off of <36 is in line with the US CDC Meningitis Laboratory protocol used by the Invasive Bacterial-Vaccine Preventable Disease (IB-VPD) surveillance network in West and Central Africa [18]. This cut-off excludes specimens that are in the equivocal range of detection by TaqMan real-time PCR [19]. Applying this cut-off, the sensitivity of the DBS assay increased from 89.1% to 100% as all 15 of the Nm positive specimens missed by the DBS assay had CTs ≥ 36 (Table 1). Likewise, the sensitivity of the DCS assay among specimens with an analytic cut-off of CT < 36 increased to 97.6% as 5/8 of the Nm meningeal infections missed by the DCS assay had CTs ≥ 36. All three false positives detected using the dried spot assay had CTs ≥ 36, hence, applying this cut-off, the specificity of both the DBS and the DCS assay was 100%.

### 2.2. Predictive Values of the Dried Spot Assay in Outbreak Scenarios

**Figure 1:**
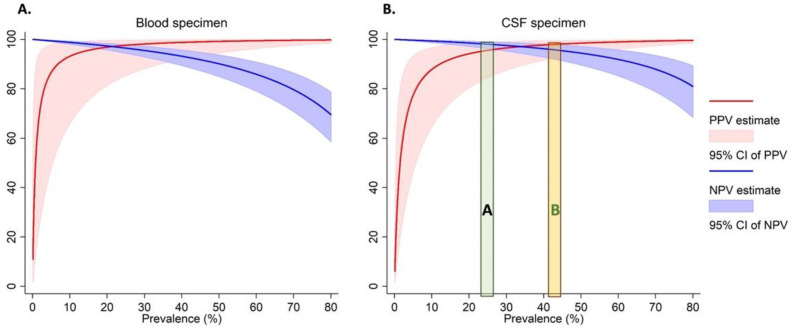
The Positive and negative predictive values for the detection of Nm in dried blood (**A**) and CSF (**B**) specimens. The green area labelled “A” represents the prevalence of *N. meningitidis* among tested specimens observed among previous meningococcal outbreaks in Niger [5] and The Gambia [20]. The orange area labelled “B” represents the prevalence of *N. meningitidis* among tested specimens observed among previous meningococcal outbreaks in Nigeria [4].

As the prevalence of Nm varies across outbreaks [4,5,20], we calculated the PPV and NPV (Figure 1 and Supplemental Table S2) over a range of real-world prevalences ranging from 20–80%. For instance, when we estimated an Nm prevalence of 40% among suspected cases as observed during the 2017 meningitis outbreak in Zamfara, Nigeria [4], the PPVs for both the DBS and DCS assays were 97% for Nm. This means that during similar Nm outbreaks, nearly all positive cases identified by the dried spot assay will be true positives. While the NPVs for both blood and CSF were high (>90%) for Nm prevalence below 50%, they tapered off at high prevalences for both specimen types. This means that at these higher prevalences, 10% to 30% of the specimens which test negative by the dried spot assay may be true cases. However, in other previous meningococcal meningitis outbreaks, e.g., NmC in Niger [5] and NmW in The Gambia [20], Nm prevalence among tested specimens was around 25% (Figure 1).

### 2.3. Genogrouping Using the Dried Spot Assay

Among the 276 positive specimens randomly selected for this study, the distribution of Nm genogroups was 76.0%, 4.3%, 3.3% and 14.5% for NmB, NmC, NmY and NmW, respectively (Table 2). A genogroup was not identified for five specimens (1.8%) using the gold standard assay performed on liquid specimen extracts. The overall agreement for meningococcal genogroups between the liquid and dried filter paper spot analysis was 81.9% (Table 2). The dried spot assay genogrouped 84.8%, 83.3%, 77.5% and 66.7% of the NmB, NmC, NmY and NmW cases, respectively. Applying the more stringent analytic cut-off of CT < 36 increased the overall agreement between the liquid and dried spot Nm genogroups to 90.1% (Table 2). Applying this cut-off, all NmC (*n* = 9) and NmW (*n* = 5) infections were identified by the dried spot assay. Likewise, 97.0% (165/170) and 96.9% (31/32) of NmB and NmY infections were correctly identified using the dried spot analysis. The dried spot analysis identified a genogroup in 4 specimens for which a genogroup was not identified using the gold standard assay. Additionally, one NmY and three NmW cases were identified as NmB infections using the dried spot assay (Table 2).

### 2.4. Whole Genome Sequencing

In an exploratory analysis, we performed targeted DNA enrichment and whole genome sequencing on DBS (*n* = 4) and DCS (*n* = 6) that had high meningococcal loads (CT ≤ 24) in the liquid specimen analysis. The genogroups identified in the liquid analysis of the specimens selected for sequencing were NmY, NmC and NmB. We obtained high-quality sequences from all ten specimens with total genome lengths of 2.06–2.15 Mbp consistent with the genome size of Nm and genome fraction of 86% against the reference genome (Table 3). A full multi-locus sequence typing (MLST) sequence type (ST) was obtained for all ten Nm strains which belonged to STs 1655, 11, 23, 485, 46 and 1423. We also obtained full genotyping (PorA and FetA) for 9 out of 10 of the specimens. Beta-lactamase resistance genes were detected in 2 out of 10 of the specimens (Table 4).

## 3. Discussion

Dried spots of blood and CSF specimens may provide a practical approach to circumvent the challenges faced in the transport and storage of diagnostic samples in low-resource settings. Leveraging well-characterised clinical specimens archived at the UKHSA Meningococcal Reference Unit, we have shown the utility of blood and CSF dried filter paper spot assays which use a crude DNA extraction method for the detection, genogrouping and genomic sequencing of Nm.

**Figure 2:**
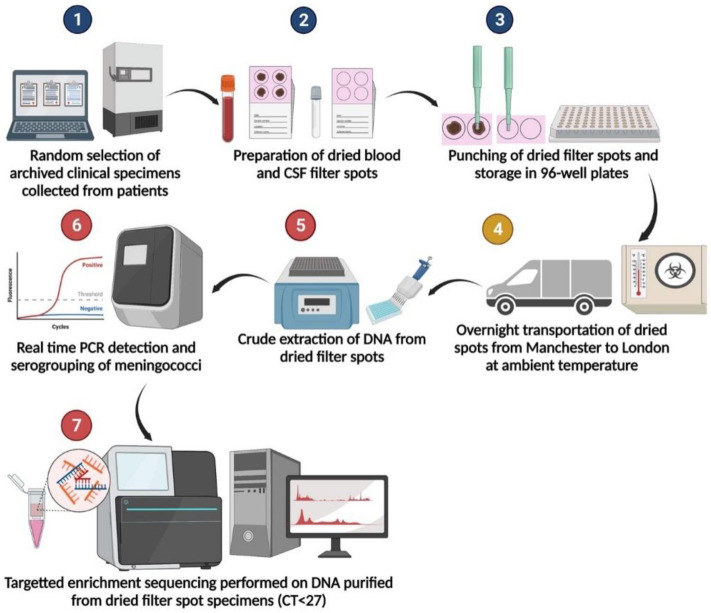
Specimen processing flow diagram. Steps 1–3 were performed at the Meningococcal Reference Unit in Manchester. Step 4 indicates shipment via courier by road. Steps 5–7 were performed at University College London.

Our results demonstrated that the specimens are stable when transported at ambient temperature overnight, an added benefit in field settings (Figure 2). This study provides valuable additional evidence for the potential benefits of using dried blood and CSF to improve case ascertainment of IMD in resource-limited settings.

At least two previous studies demonstrated that DCS assays targeting bacterial pathogens can be highly sensitive and specific. Fukasawa and colleagues tested the performance of a DCS assay using FTA^TM^ cards and specimens collected as part of a national surveillance programme in Brazil [21]. Of the 213 dried CSF specimens, they tested for Nm, *S. pneumoniae* and *Haemophilus influenzae*, the specificities were 100% and the sensitivities ranged from 96 to 100% across all three pathogens. Likewise, De Vitis and colleagues found that among 44 CSF specimens for which a bacterial infection was confirmed by qPCR of the liquid specimen, 41 (93.2%) were positive by testing on dried filter paper [22]. Our study provides further evidence that compared to real-time PCR on liquid specimens, dried filter spot assays are highly specific and sensitive for the detection of Nm in CSF. Our dried CSF assay for meningococcal detection had comparable specificity of 99% and a sensitivity of 94%.

Unlike DSC assays, the performance of DBS assays for the detection of bacterial infections varies substantially across studies. Among febrile children in Nigeria, dried blood spot assays had sensitivities ranging from 57% to 63% for the detection of *S. pneumoniae* compared to culture and qPCR on liquid specimens [14,23]. Fukasawa and colleagues reported sensitives of 73%, 88% and 100% for the detection of Nm, *S. pneumoniae* and *H. influenzae*, respectively, in serum dried spots using FTA^TM^ cards [22]. False negatives in DBS testing may result from the presence of inhibitors in blood such as immunoglobulin, haemoglobin, and metal ions [24,25].

Advances in PCR-approaches have led to the development of DNA polymerases and PCR reagents whose activity can overcome the presence of PCR inhibitors and allow amplification of the target genes directly from clinical specimens without the need to purify nucleic acids. The real-time PCR mastermix we used neutralises PCR inhibitors and showed high performance in DBS specimens. Another innovation in our dried spot assay for the detection of Nm is the use of a crude DNA extraction method which is affordable (<$0.30 per sample), has high throughput (simultaneous processing of 96 samples and controls) and is rapid (~30 min).

Traditional nucleic acid purification kits typically have multiple manipulation steps which increase the risk of cross-contamination and use large quantities of consumables such as plastic pipette tips. Our dried blood spot assay using the crude DNA extraction method showed a high sensitivity (89%) and specificity (99%) for the detection of Nm in dried blood spots. These results are comparable to the sensitivity and specificity of 90.3% and 99%, respectively reported by De Vitis and colleagues for their dried spot assay which utilised the MagCore automated NucleicAcid Extractor with MagCore Nucleic Acid Tissue Extraction Kit [22]. Crude DNA extraction from blood and other clinical specimens therefore could be a viable alternative to traditional extraction kits and make dried spot assays for the diagnosis of bacterial meningitis more accessible in resource-limited settings.

The emergence of hypervirulent NmC, NmW, NmY and NmX strains with epidemic potential reinforces the need for close monitoring and surveillance of circulating serogroups and genotypes [4,5,6,7]. Hence, an ideal dried spot assay for the detection of IMD should also support accurate serogrouping and genomic sequencing analysis. Using our dried spot assay, we determined the serogroup in 81.9% of the confirmed meningococcal infections. All the blood and CSF specimens for which a serogroup could not be determined using the dried spot assay had lower bacterial loads (CT > 32). We also demonstrated that the whole genomes of Nm strains can be successfully sequenced from DBS and DCS using targeted DNA enrichment. We obtained near full-length genome sequences (~86%) for all ten specimens tested (the differences likely reflected variability in the accessory genome of the reference sequence and our strains) which allowed determination of the full sequence type, clonal complex, presence of antimicrobial resistance and other Nm genotyping. To our knowledge, this is the first study to demonstrate that whole-genome sequencing of Nm from blood and CSF-dried filter paper spots can be performed successfully. Our findings support the use of dried filter spots as a robust and feasible alternative to liquid specimens for investigations requiring whole-genome sequencing in resource-limited settings.

The US CDC Meningitis Laboratory recommends categorising specimens positive for Nm if the detection CT < 35, equivocal if the detection CT is 36–40, and negative if the detection CT > 40 [19]. The WHO Invasive-Bacterial Vaccine-Preventable Disease (IB-VPD) surveillance network in West and Central Africa follows these US CDC guidelines, and therefore, adopts this conservative cut-off [18]. Hence, we explored the performance of our dried spot assay using a conservative cut-off for positivity of CT < 36 for the liquid specimens. After applying the cut-off of CT < 36, the sensitivity of the DBS assay improved to 100% and 97.6% for the DCS assay. We also observed that genogrouping of the meningococci using the dried spot assay improved from 82% to 90% when equivocal specimens with CT ≥ 36 were excluded. These findings suggest that the reliability and performance of these blood and CSF dried spot methods improves substantially with increasing bacterial loads. 

Although we attempted to simulate field scenarios transporting samples across laboratories by road, the evaluation of this assay will need to be performed in the African meningitis belt where various epidemiologic, climatic, logistic, and technical factors could affect the assay’s performance. For instance, the leading meningococcal serogroup in the UK is NmB, however, NmB is very rare in the African meningitis belt where other meningococcal serogroups such as NmC, NmW, NmY and NmX dominate [4,18]. Another important limitation is the small numbers per Nm serogroup which limited the precision of the agreement analysis, particularly for NmY, NmC and NmW serogroups.

We have evaluated the performance of DBS and DCS assays for the detection and typing of Nm, however, their utility could be extended by including the detection and typing of other important causes of acute bacterial meningitis such as *S. pneumoniae* and *H. influenzae*. The targeted DNA enrichment and whole genome sequencing method used to sequence Nm from dried clinical specimens may be prohibitively expensive in low-resource settings. Hence, the development of alternative approaches for targeted sequencing of pathogens from dried spots needs to be explored. One such approach could be selective whole-genome amplification (SWGA) which enables the efficient enrichment, sequencing, and de novo assembly of Nm from clinical specimens [26,27].

In conclusion, dried spot assays could increase the diagnostic yield of confirmed IMD in Africa while reducing the need for cold chains or large volumes of clinical specimens. This approach has the potential to hasten essential responses during public health emergencies in Africa.

## 4. Materials and Methods

### 4.1. Clinical Specimens

We evaluated the performance of a dried blood and CSF filter spot assay compared to standard molecular diagnosis of meningococcal infections from liquid specimens. All clinical samples were initially submitted to the UKHSA MRU for diagnosis of IMD between 2007 and 2020. During the initial investigation, DNA was extracted from the samples using a column-based extraction protocol utilising a Qiagen BioRobot MDx (Qiagen, UK). Extracts were then tested using meningococcal TaqMan PCR assay targeting the *ctrA* and *siaD* capsular genes allowing species confirmation and genogroup determination [28,29]. The UKHSA MRU stores all meningococcal-positive clinical material under a Human Tissue Act research license. 

Assuming a sensitivity of 90% for the detection of Nm with 5% precision, a minimum number of 138 blood specimens and 138 CSF specimens that were positive for Nm using the gold standard (real-time PCR performed on liquid specimens) were required for the study. Likewise, for the specificity, 138 blood and 138 CSF samples negative for Nm by the gold standard were required to detect a specificity of 90% for the dried spot assay with 5% precision. Therefore, a total of 552 blood and CSF specimens were randomly selected from among the archived specimens using the above criteria. Specimens with low sample volumes <0.05 mL and those without real-time PCR results for Nm detection were excluded.

### 4.2. Preparation of Dried Spots

Archived whole blood and CSF specimens stored at −80 °C were gently thawed and vortexed before 50 μL of each specimen was inoculated onto a Whatman^®^ 903 protein saver card at the UKHSA MRU. The inoculated DBS and DCS specimens were dried for one hour and then stored in zip-lock bags with a desiccant at ambient temperature (18–25 °C). Following overnight storage, three 3 mm punches of each dried specimen were prepared and placed in 96-well plates in a randomised distribution. To avoid cross-contamination, the punches were carefully cleaned using 70% ethanol and sterile molecular grade water between specimens. The sample flow is summarised in Figure 2.

### 4.3. Genomic DNA Extraction from Dried Specimens

The DBS and DCS specimens prepared at the UKHSA MRU in Manchester were shipped in bulk to University College London (UCL) for molecular analysis via courier at ambient temperature in sealed 96-well plates. The analysts were blinded to the allocation of Nm positive and negative specimens across each plate. To reduce costs and extraction times, a crude genomic DNA extraction method, QuantaBio Extracta DBS (Beverly, MA, USA), was adapted for the purification of genomic DNA from DBS and DCS specimens for this assay.

To reduce the cost, resources and time required for genomic DNA extraction, we optimised this assay using a crude extraction procedure as follows. Three 3 mm filter paper punches were resuspended in 0.1 mL of the Extracta DBS reagent and centrifuged at 3900× *g* for 5 min. Following removal of the supernatant, the punches were resuspended in 0.05 mL of the Extracta DBS reagent and incubated at 95 °C for 30 min. The crude genomic DNA was then stored at 4–8 °C.

### 4.4. Real-Time PCR on Dried Specimens

Nm was detected using species-specific real-time PCR assays targeting the capsule transport to cell surface gene *ctrA* and the copper- and zinc-cofactored superoxide dismutase gene *sodC* as described previously [19,30,31,32]. Nm genogrouping was performed by real-time PCR assays as previously described [19] which targeted the capsule biosynthesis genes: *siaDb*/*csb*, *siaDc*/*csc*, *siaDw*/*csw and siaDy*/*csy* genes for NmB, NmC, NmW and NmY, respectively.

The standard real-time PCR components were 1x QuantaBio PerfeCTa qPCR Toughmix (Beverly, MA, USA), the forward and reverse primers, probe and 5 μL of the genomic DNA with the volume brought up to 25 μL with molecular grade water. The primer and probe sequences and final concentrations in the real-time PCR reactions are outlined in Supplemental Table S1. The real-time PCR assays were run on the Eppendorf Mastercycler^®^ ep RealPlex system (Eppendorf, Hamburg, Germany). After the initial denaturation at 95 °C for 5 min, there were 40 cycles at 95 °C for 15-s and 60 °C for 60 s. Specimens with Cycle Threshold (CT) values ≤ 40 were considered positive. All PCRs were run with non-template (NTC) and positive controls.

### 4.5. Targeted DNA Enrichment and Whole-Genome Sequencing from Dried Specimens

Genomic DNA was extracted from four 3 mm filter paper punches of each DBS and DCS specimen using the QIAamp^®^ DNeasy Blood and Tissue Kit (Qiagen, Manchester, UK) following the manufacturer’s protocol with minor modifications. Each specimen was eluted in 150μL of the elution buffer.

The purified DNA samples were stored at −20 °C and submitted to the UCL Pathogen Genomics Unit (PGU) for RNA-bait targeted enrichment and whole-genome sequencing of *N. meningitis* as described previously [33]. Adapter sequences were trimmed from the reads using cutadapt v3.5 [34]. To remove the host-associated reads, we mapped the sequencing reads of each isolate against the human reference genome GRCh38 (hg38) using Bowtie2 v2.3.0. We then filtered out read pairs that did not concordantly map to the reference genome (by specifying the—un-conc option in Bowtie2 v2.3.0). We then removed duplicate reads resulting from PCR artefacts using PRINSEQ v0.20.4 [35]. The reads were assembled using SPAdes v3.13.0 genome assembly to generate contiguous sequences for downstream analysis. The statistics for each generated draft assembly were calculated using QUAST v5.0.2 [36]. We used the *N. meningitidis* Z2491 complete reference genome sequence (GenBank accession: NC_003116) to assess the percentage genome coverage [36]. The ST or clone was inferred based on the MLST scheme for *Neisseria* species by scanning the draft assembled genomes using the “mlst” Perl script (https://github.com/tseemann/mlst (accessed on 3 December 2021)) using a database available from PubMLST [37].

### 4.6. Statistical Analysis

The results for the detection of Nm using the dried filter spot assay and the gold standard were coded as binary variables (positive or negative). Sensitivity for the Nm detection was calculated as the proportion of samples returning a positive result when tested using the dried filter spot assay divided by the total number of samples positive based on the gold standard. Exact binomial confidence intervals for each sensitivity estimate were computed. A similar approach was used for the specificity estimate with the exception that specificity for the Nm detection assay was calculated as the proportion of samples returning a negative result when tested using the new assays divided by the total number of samples negative by the gold standard analysis.

The positive predictive value (PPV) and negative predictive value (NPV) for the Nm detection assay were estimated using the sensitivity and specificity estimates from the validity analysis, for a range of plausible real-world disease prevalence values. The PPV is defined as the proportion of samples with disease (as measured by a gold standard), given a positive test result using the new assay. The NPV was defined as the proportion of specimens without Nm (as measured by a gold standard), given a negative test result using the dried spot assay.

The overall percentage agreement and Cohen’s Kappa coefficient were estimated to evaluate the concordance between genogrouping from the dried filter spot and PCR assays for samples positive on PCR. The correlation between the CT values for the dried filter spot and liquid assays was also assessed using the Pearson correlation coefficient including and excluding potential outliers.

All analyses were implemented using Stata 14.2.

## Figures and Tables

**Table 1:** Sensitivity and specificity of dried spot assay for blood and CSF specimens.

ALL SPECIMENS								
Specimen Type	Dried Spot Extract	Liquid Specimen Extract ^#^			*p* ^a^	% of Agreement (95% CI) ^b^	Sensitivity (95% CI) ^b^	Specificity (95% CI) ^b^
		Positive	Negative	Total				
Blood	Positive	123	1	124	<0.001	94.2 (90.8–96.7)	89.1 (82.7–93.8)	99.3 (96.0–99.9)
	Negative	15	137	152				
	Total	138	138	276				
CSF	Positive	130	2	132	0.11	96.4 (93.5–98.0)	94.2 (88.9–97.5)	98.6 (94.9–99.8)
	Negative	8	136	144				
	Total	138	138	276				
SPECIMENS WITH CT < 36 IN LIQUID QPCR								
Specimen Type	Dried spot extract	Liquid specimen extract ^#^			*p* ^a^	% of agreement (95% CI) ^b^	Sensitivity (95% CI) ^b^	Specificity (95% CI) ^b^
		Positive	Negative	Total				
Blood	Positive	106	0	106	-	-	-	-
	Negative	0	0	0				
	Total	106	0	106				
CSF	Positive	124	0	124	0.25	97.6 (93.3–99.5)	97.6 (93.3–99.5)	-
	Negative	3	0	3				
	Total	127	0	127				

^a^ Exact McNemar test. ^b^ Exact binomial 95% confidence intervals. ^#^ Used as the gold standard.

**Table 2:** Agreement between genogrouping from dried spot extracts and liquid specimen extracts.

ALL QPCR POSITIVE SPECIMENS (*n* = 276)								
Dried filter paper spot analysis	Liquid analysis							% of agreement: 81.9% Kappa coefficient: 0.61 *p* < 0.001
	Genogroup	B	C	W	Y	Negative	Total	
	B	178 (84.8)	0 (0)	3 (33.3)	1 (2.5)	3 (60.0)	185 (100)	
	C	0 (0)	10 (83.3)	0 (0)	0 (0)	0 (0)	10 (100)	
	W	0 (0)	0 (0)	6 (66.7)	0 (0)	0 (0)	6 (100)	
	Y	0 (0)	0 (0)	0 (0)	31 (77.5)	1 (20.0)	32 (100)	
	Negative	32 (15.2)	2 (16.7)	0 (0)	8 (20.0)	1 (20.0)	43 (100)	
	Total	210 (100)	12 (100)	9 (100)	40 (100)	5 (100)	276 (100)	
ALL QPCR POSITIVE SPECIMENS WITH CT < 36 (*n* = 233)								
Dried filter paper spot analysis	Liquid analysis							% of agreement: 90.6% Kappa coefficient: 0.77 *p* < 0.001
	Genogroup	B	C	W	Y	Negative	Total	
	B	165 (92.7)	0 (0)	2 (28.6)	0 (0)	3 (60.0)	170 (100)	
	C	0 (0)	9 (90.0)	0 (0)	0 (0)	0 (0)	9 (100)	
	W	0 (0)	0 (0)	5 (71.4)	0 (0)	0 (0)	5 (100)	
	Y	0 (0)	0 (0)	0 (0)	31 (93.9)	1 (20.0)	32 (100)	
	Negative	13 (7.3)	1 (10.0)	0 (0)	2 (6.1)	1 (20.0)	17 (100)	
	Total	178 (100)	10 (100)	7 (100)	33 (100)	5 (100)	233 (100)	

**Table 3:** Outcome of targeted DNA enrichment and whole genome sequencing of *N. meningitidis* from dried clinical specimens.

Strain	PubMLST ID	Source	Geno-Group	Reference Genome Length	Genome Fraction (%)	Largest Alignment	Total Aligned Length	Number of Contigs	Largest Contig Size	Total Length	N50	GC (%)
DS101	122167	CSF	Y	2,184,406	87.24	70,343	1,914,699	121	104,093	2,088,119	17,857	52.06
DS103	122168	CSF	C	2,184,406	86.07	82,895	1,889,630	95	161,253	2,121,246	45,825	51.79
DS104	122169	CSF	Y	2,184,406	87.53	80,073	1,921,171	93	104,803	2,128,519	36,260	51.99
DS105	122170	CSF	Y	2,184,406	87.78	70,344	1,926,030	110	104,975	2,079,216	33,372	52.05
DS106	122171	CSF	Y	2,184,406	87.58	70,344	1,915,987	107	105,166	2,093,998	36,146	52.05
DS107	122172	CSF	B	2,184,406	86.47	131,466	1,900,683	86	159,697	2,124,689	56,415	51.72
DS109	122173	Blood	Y	2,184,406	87.38	70,343	1,912,659	102	101,432	2,064,702	36,768	52.02
DS110	122174	Blood	B	2,184,406	86.28	86,261	1,892,448	97	92,791	2,102,530	39,425	51.84
DS111	122175	Blood	B	2,184,406	86.17	74,489	1,891,092	129	81,727	2,140,198	33,412	51.83
DS112	122176	Blood	B	2,184,406	86.04	115,418	1,894,923	108	172,619	2,151,200	56,615	51.64

**Table 4:** Genotyping of *N. meningitidis* sequenced from dried clinical specimens through targeted DNA enrichment.

ID	ST	AbcZ	Adk	AroE	FumC	Gdh	PdhC	Pgm	AMR *	PorA_VR1	PorA_VR2	FetA_VR
DS101	1655	12	5	18	9	11	9	17		5-1	10-1	F4-1
DS103	11	2	3	4	3	8	4	6	penA	5-1	10-8	F3-6
DS104	1655	12	5	18	9	11	9	17		5-1	10-1	F4-1
DS105	23	10	5	18	9	11	9	17	penA	5-1	10-4	F4-1
DS106	23	10	5	18	9	11	9	17		5-1	10-4	F4-1
DS107	32	4	10	5	4	6	3	8		7	16	F3-3
DS109	1655	12	5	18	9	11	9	17		-	-	F4-1
DS110	485	3	6	9	5	8	6	9		12-1	16	f1-5
DS111	46	3	6	9	5	3	6	9		7-2	4	F1-5
DS112	1423	9	5	9	9	9	6	2		17-1	23	F1-5

* AMR: Antimicrobial resistance genes.

## Data Availability

The *N. meningitidis* genome assemblies generated during this study are available on PubMLST (https://pubmlst.org/organisms/neisseria-spp (accessed on 3 December 2021)) and the PubMLST IDs for each specimen are listed in Table 3. Materials (clinical and biological specimens) will be shared upon request for health research provided there are sufficient quantities, appropriate agreements, and ethical approvals in place. Further information and requests for resources and reagents should be directed to and will be fulfilled by the lead contacts Brenda A. Kwambana-Adams (Brenda.Kwambana@ucl.ac.uk) and Ray Borrow Ray.Borrow@ukhsa.gov.uk.

## Supplementary Materials

The supporting information can be downloaded at: https://www.mdpi.com/article/10.3390/ijms231911879/s1. Reference [19] is cited in the supplementary materials.
