# Supplementary material for: Evaluation of Dried Blood and Cerebrospinal Fluid Filter Paper Spots for Storing and Transporting Clinical Material for the Molecular Diagnosis of Invasive Meningococcal Disease
Source: Int J Mol Sci. 2022 Oct 6;23(19):11879. doi: 10.3390/ijms231911879 (PMC9569512; doi:10.3390/ijms231911879)
Supplement: Supplementary file 1 [file ijms-23-11879-s001.zip › ijms-1936096-supplementary.pdf]

Title:

Evaluation of Dried Blood and Cerebrospinal Fluid Filter Paper Spots for Storing and Transporting Clinical Material for the Molecular Diagnosis of Invasive Meningococcal Disease

Supplemental Table S1: Real time PCR assays for the detection of *N. meningitidis* in dried blood and CSF specimens.

**Target**	**Primer or Probe Name**	**Real-time Primers and Probes Nucleotide Sequence (5′ to 3′)**	**Final Conc (nM)**	**Probe Modifications**
N. meningitidis				
ctrA	F753	TGTGTTCCGCTATACGCCATT	300	
	R846	GCCATATTCACACGATATACC	600	
	Pb820i	AACCTTGAGCAA"T"CCATTTATCCTGACGTTCT	200	5′ FAM, BHQ1 on "T", 3′ SpC6
sodC	F351	GCACACTTAGGTGATTTACCTGCAT	300	
	R478	CCACCCGTGTGGATCATAATAGA	300	
	Pb387	CATGATGGCACAGCAACAAATCCTGTTT	200	5′ FAM, 3′ BHQ1
**Nm A**	F2531	AAAATTCAATGGGTATATCACGAAGA	900	
sacB	R2624	ATATGGTGCAAGCTGGTTTCAATAG	600	
	Pb2591i	CTAAAAG"T"AGGAAGGGCACTTTGTGGCATAAT	300	5′ FAM, BHQ1 on "T", 3′ SpC6
**Nm B**	F737	GCTACCCCATTTCAGATGATTTGT	900	
synD	R882	ACCAGCCGAGGGTTTATTTCTAC	300	
	Pb839i	AAGAGATGGGYAACAAC"T"ATGTAATGTCTTTATTT	200	5′ FAM, BHQ1 on "T", 3′ SpC6
**Nm C**	F478	CCCTGAGTATGCGAAAAAAATT	300	
synE	R551	TGCTAATCCCGCCTGAATG	300	
	Pb495i	TTTCAATGC"T"AATGAATACCACCGTTTTTTTGC	300	5′ FAM, BHQ1 on "T", 3′ SpC6

**Nm W**	F857	TATTTATGGAAGGCATGGTGTATG	300	
synG	R964	TTGCCATTCCAGAAATATCACC	100	
	Pb907i	AAATATGGAGCGAATGATTACAG TAACTATAATGAA	200	5′ FAM, BHQ1 on "T", 3′ SpC6
**Nm X**	F173	TGTCCCCAACCGTTTATTGG	900	
xcbB	R237	TGCTGCTATCATAGCCGCC	900	
	Pb196	TGTTTGCCCACATGAATGGCGG	100	5′ FAM, 3′ BHQ1
**Nm Y**	F787	TCCGAGCAGGAAATTTATGAGAA TAC	900	
synF	R929	TTGCTAAAATCATTCGCTCCATAT	300	
	Pb1099i	TATGGTG"T"ACGATATCCCTATCC TTGCCTATAAT	300	5′ FAM, BHQ1 on "T", 3′ SpC6

Assay adapted from the US Centers for Disease Control and Elimination (US CDC). The protocol was previously published[1] and further details are available by following this link https://www.cdc.gov/meningitis/lab-manual/chpt10-pcr.html

Supplemental Table S2: PPV and NPV of dried spot assay stratified by specimens' type.

**Specimen**	**Prevalence (%)**	**PPV (95% CI)**	**NPV (95% CI)**
Blood	20	96.9 (81.3-99.5)	97.3 (95.8-98.3)
	30	98.1 (88.2-99.7)	95.5 (93.0-97.2)
	40	98.8 (92.1-99.8)	93.2 (89.5-95.7)
	50	99.2 (94.6-99.9)	90.1 (85.0-93.6)
	60	99.5 (96.3-99.9)	85.9 (79.1-90.8)
	70	99.7 (97.6-99.9)	79.7 (70.8-86.3)
	80	99.8 (98.6-99.9)	69.5 (58.6-78.6)
CSF	20	94.2 (80.4-98.5)	98.6 (97.2-99.3)
	30	96.5 (87.6-99.1)	97.5 (95.3-98.7)

40	97.7 (91.6-99.4)	96.2 (92.9-98.0)
50	98.5 (94.3-99.6)	94.4 (89.7-97.1)
60	99.0 (96.1-99.7)	91.9 (85.3-95.7)
70	99.3 (97.5-99.8)	87.9 (78.8-93.5)
80	99.6 (98.5-99.9)	81.0 (68.4-89.3)

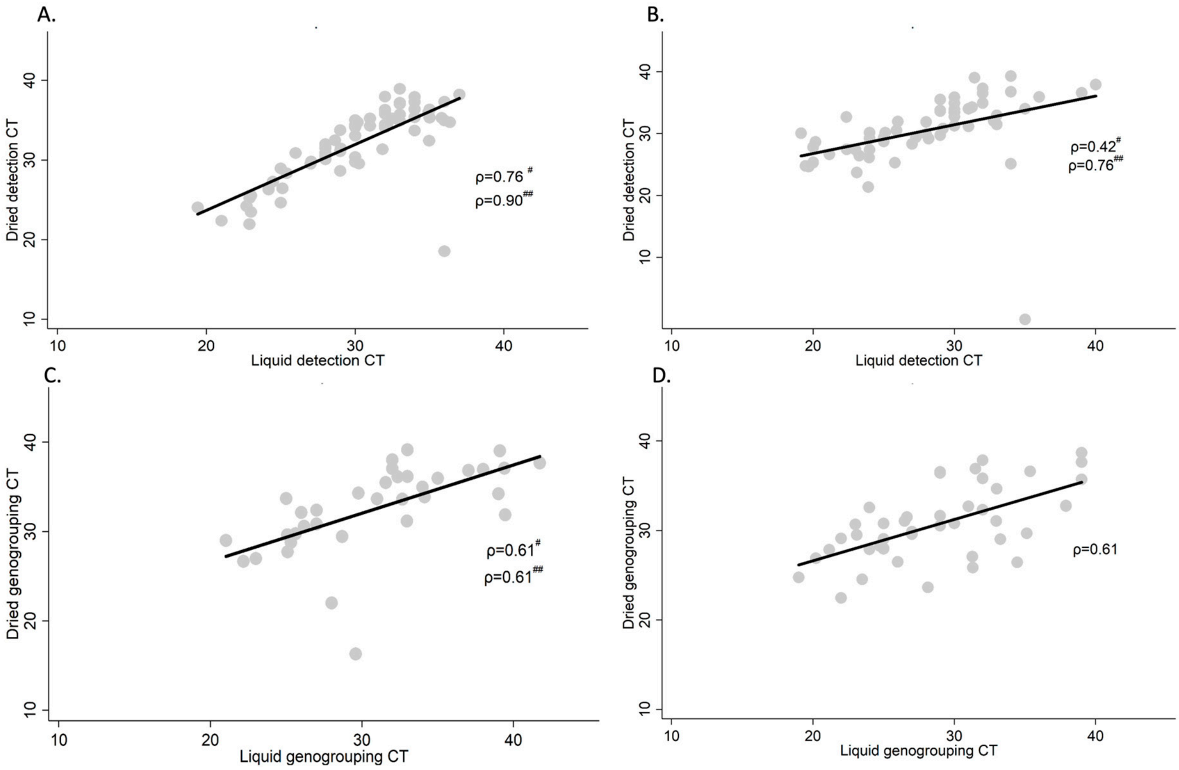

Supplemental Figure S1. Correlations between liquid (gold standard) and dried filter spot assays for the detection and genogrouping of *N. meningitidis* infections.

#Correlattion coefficient with outliers
##Correlattion coefficient without outliers

Reference

1. Vuong J, Collard JM, Whaley MJ, Bassira I, Seidou I, Diarra S, Ouedraogo RT, Kambire D, Taylor TH, Jr., Sacchi C, Mayer LW, Wang X. Development of Real-Time PCR Methods for the Detection of Bacterial Meningitis Pathogens without DNA Extraction. PLoS One **2016**; 11(2): e0147765. 10.1371/journal.pone.0147765
